# Improvement of AlGaN/GaN High-Electron-Mobility Transistor Radio Frequency Performance Using Ohmic Etching Patterns for Ka-Band Applications

**DOI:** 10.3390/mi15010081

**Published:** 2023-12-30

**Authors:** Ming-Wen Lee, Cheng-Wei Chuang, Francisco Gamiz, Edward-Yi Chang, Yueh-Chin Lin

**Affiliations:** 1 International College of Semiconductor Technology, National Yang Ming Chiao Tung University (NYCU), Hsinchu City 30010, Taiwan; ericlmw.st06@nycu.edu.tw (M.-W.L.); kaszxc861229@gmail.com (C.-W.C.); edc@nycu.edu.tw (E.-Y.C.); 2 Department of Electronics and Computer Technology, University of Granada, 18014 Granada, Spain; fgamiz@ugr.es; 3 Institute of Microengineering and Nanoelectronics, University Kebangsaan Malaysia, Bangi 43600, Malaysia

**Keywords:** aluminum gallium nitride, etching, HEMTs, large signal, noise figure, ohmic contacts, radio frequency, small signal

## Abstract

In this paper, AlGaN/GaN high-electron-mobility transistors (HEMTs) with ohmic etching patterns (OEPs) “fabricated to improve device radio frequency (RF) performance for Ka-band applications” are reported. The fabricated AlGaN/GaN HEMTs with OEP structures were used to reduce the source and drain resistances (*R_s_* and *R_d_*) for RF performance improvements. Within the proposed study using 1 μm hole, 3 μm hole, 1 μm line, and 3 μm line OEP HEMTs with 2 × 25 μm gate widths, the small signal performance, large signal performance, and minimum noise figure (*NF_min_*) with optimized values were measured for 1 μm line OEP HEMTs. The cut-off frequency (*f_T_*) and maximum oscillation frequency (*f_max_*) value of the 1 μm line OEP device exhibited optimized values of 36.4 GHz and 158.29 GHz, respectively. The load–pull results show that the 1 μm line OEP HEMTs exhibited an optimized maximum output power density (P_out, max_) of 1.94 W/mm at 28 GHz. The 1 μm line OEP HEMTs also exhibited an optimized *NF_min_* of 1.75 dB at 28 GHz. The increase in the contact area between the ohmic metal and the AlGaN barrier layer was used to reduce the contact resistance of the OEP HEMTs, and the results show that the 1 μm line OEP HEMT could be fabricated, producing the best improvement in RF performance for Ka-band applications.

## 1. Introduction

With the growth of the Internet of Things (IoTs), artificial intelligence (AI), and the increasing demand for high-speed consumer electronics such as smart phones, smart homes, and unmanned aerial vehicles (UAV), lower-frequency system bandwidths for data transmission have become congested [1,2,3,4]. As a result, Ka-band systems have emerged in fifth-generation (5G) and beyond 5G (B5G) systems to increase spectrum allocations and data rates, and to reduce antenna sizes [5]. Power amplifiers (PAs) and low noise amplifiers (LNAs) in wireless communication circuits and their transistors are especially crucial when it comes to enhancing efficiency, gaining flatness, and lowering noise over a wide-frequency band [6]. Silicon-based transistors, such as complementary metal–oxide semiconductor field-effect transistors (CMOS FETs), can be used in radio frequency (RF) transceiver circuits given their low costs and high yield, but they suffer from low power gain, a short channel effect, and saturation effects due to scaling [7,8]. III–V-based transistors, such as AlGaAs/GaAs and AlGaN/GaN high-electron-mobility transistors (HEMTs), are also used in high-frequency PAs and LNAs, but GaAs-based HEMTs suffer from low voltage operation, low power per unit, and low power efficiency due to the small energy bandgap and low breakdown voltage [9,10,11]. On the other hand, GaN-based HEMTs have demonstrated stronger frequency response, higher power-added efficiency (PAE), and better power performance, so are suitable for 5G and B5G systems, ranging from sub-6 GHz to Ka band, due to the high breakdown voltage, high saturation current, and low-frequency noise characteristics [12,13,14,15,16,17].

Nevertheless, parasitic resistance builds up at ultra-high frequencies for GaN HEMTs due to their high operation voltages, which reduces the overall device performance, such as current density, RF power, and PAE. Solutions have been proposed to reduce the parasitic influences of GaN-based HEMTs through barrier layer recessing, ohmic regrowth, and n-type doping to lower the source and drain resistances (*R_s_* and *R_d_*) for direct current (DC) characteristic improvements [18,19]. In advance, researchers have reported simulated and experimental results regarding contact resistivity improvements using several ohmic recessing patterns to increase the current paths and device saturation current density [20,21,22,23,24,25,26,27].

This study further compared the DC, RF small signal, RF large signal, and RF noise performance of different ohmic etching patterns (OEPs) for Ka-band applications and to design an optimized OEP structure with lower source and drain resistances, higher saturation current density, better RF power performance, and a lower high-frequency noise. The optimized OEP device with a 1 μm line pattern demonstrated the lowest contact resistance, highest small signal and large signal performance, and the smallest minimum noise figure (*NF_min_*) at the Ka band among the four OEP structures designed in this study.

## 2. Materials and Methods

**Figure 1:**
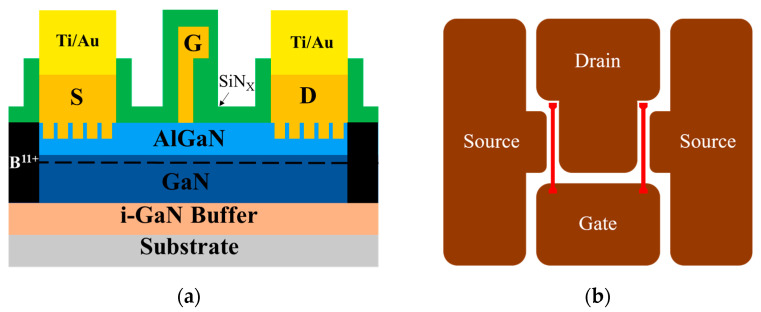
Schematic graph of the OEP AlGaN/GaN HEMT with the (**a**) cross-section view and the (**b**) top view.

The AlGaN/GaN HEMTs were fabricated on a 4 inch GaN on SiC substrate. The epitaxial wafer was grown with a metal–organic chemical vapor deposition (MOCVD) system and consisted of an i-GaN buffer layer, a 0.9 μm unintentionally doped GaN channel layer, a 25 nm Al_0.25_Ga_0.75_N barrier layer, and a 2 nm GaN cap layer, as shown in Figure 1a. The device structure consisted of two gate fingers (red), one gate pad, two source pads, and one drain pad, as shown in Figure 1b. The epitaxial wafer was measured via Hall measurement at room temperature and showed an electron mobility of 1500 cm^2^/V·s, a sheet resistance of 280 Ω/sq, and a sheet carrier density of 1 × 10^13^/cm^2^.

**Figure 2:**
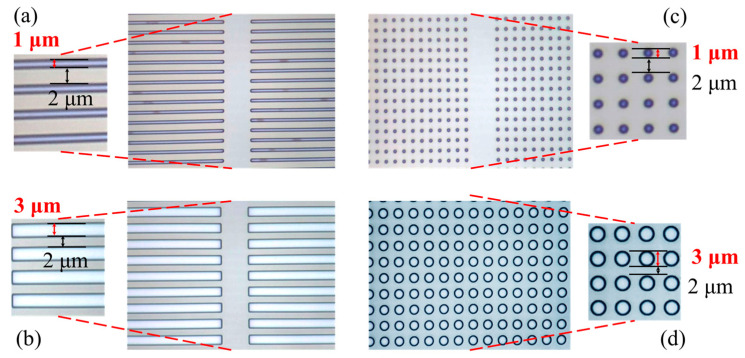
Optical microscope pictures of the developed OEPs with (**a**) 1 μm lines, (**b**) 3 μm lines, (**c**) 1 μm holes, and (**d**) 3 μm holes.

Alignment marks were fabricated first on the epitaxial wafer during the OEP process. Four different OEPs of 1 μm lines, 3 μm lines, 1 μm holes, and 3 μm holes, respectively, were then defined and transferred to the wafer by the stepper photolithography system (stepper) [12]. There are two shapes among the four OEPs, the line patterns and the hole patterns. The defined line patterns are parallel to the current flow and have widths of 1 μm or 3 μm, with lengths equal to the source and drain active area, and separations of 2 μm between the pattern edges. The defined hole patterns have diameters of 1 μm or 3 μm, for the 1 μm holes and 3 μm holes, respectively, and the hole patterns are distributed uniformly over the source and drain active area with a 2 μm separation between the pattern edges. The optical micrographs of the developed OEP structures on the epitaxial wafer with 1 μm lines, 3 μm lines, 1 μm holes, and 3 μm holes are shown in Figure 2a,b,c,d, respectively. The schematic position of the OEP structures are shown in Figure 1a with the hole pattern rather than the line pattern for clarity.

To form OEPs at the source and drain the ohmic contact area, the inductively coupled plasma-reactive ion etching (ICP-RIE) system is then used to dry etch the GaN cap layer and the AlGaN barrier layer with Cl_2_/BCl_3_ plasma. The OEPs were etched to around 10 nm above the 2-dimensional-electron-gas (2DEG) channel, which etch-stopped at the AlGaN barrier layer. The recessed depths were chosen only above 2DEG, due to the higher contact resistances measured for the devices with recessed depth below 2DEG, as shown in previous research [28]. After wafer cleaning with a diluted hydrochloric acid (HCl) solution to remove the native oxide layer [29], an ohmic metal stack of Ti/Al/Ni/Au was deposited with the e-beam evaporation system (E-gun) and annealed by the rapid thermal annealing system (RTA) at 850 °C for 30 s in N_2_ ambient. The RTA process was followed by the B^11+^ ion implantation to define the active region of the devices. After the gate length (L_g_) definition of 0.15 μm by the stepper using the 2-step photolithography process [12], the wafer was also uniformly dipped in a diluted HCl solution to remove native oxide layers before gate metal deposition [29]. Ni/Au was then deposited as the gate metal stack for Schottky contact formation and a 100 nm SiN_X_ passivation layer was deposited using the plasma enhanced chemical vapor deposition (PECVD) for moisture protection [30]. After via-opening of the SiN_X_ layer on the contact metal pads with the ICP system, thick metallization of a 2 μm Ti/Au metal stack was deposited using an E-gun after a wafer cleaning process using diluted HCl solution.

## 3. Results and Discussion

### 3.1. DC Characteristics

Transmission line modeling (TLM) was used in this study to determine the specific contact resistivity (ρ_c_) and the contact resistance (R_c_) of the epitaxial wafer with the four designed OEP structures and the results are shown in Table 1 [25,26].

The ρ_c_ has been improved from 2.73 × 10^−6^ Ω·cm^2^ to 4.04 × 10^−7^ Ω·cm^2^ and the R_c_ has been improved from 0.429 Ω·mm to 0.154 Ω·mm applying the 1 μm line OEP structure, which is the lowest value among the fabricated TLM structures with the four designed OEPs.

**Figure 3:**
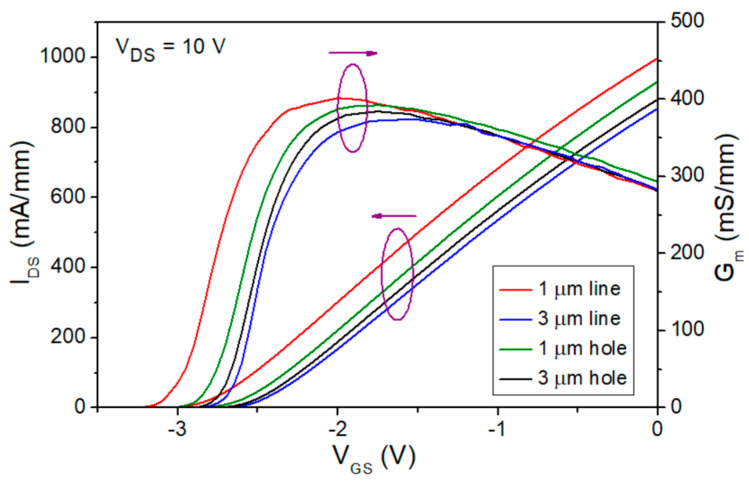
I_DS_−V_GS_ and G_m_−V_GS_ curves for the 2 × 25 μm AlGaN/GaN HEMTs with different OEPs. (Left arrow: I_DS;_ Right arrow: G_m_).

AlGaN/GaN HEMTs with the four designed OEPs were also fabricated on the same epitaxial wafer. The I_DS_−V_GS_ and G_m_−V_GS_ curves for the four fabricated OEP AlGaN/GaN HEMTs are shown in Figure 3. The gate width and source-to-drain spacing (L_SD_) for the OEP GaN HEMTs are 2 × 25 μm and 2 μm, respectively. The gate-to-drain spacing (L_GD_) of 1.25 μm, and a gate-to-source spacing (L_GS_) of 0.6 μm were designed for the devices. The peak extrinsic transconductance (G_m, peak_) of 403 mS/mm and the drain-to-source saturation current (I_DSS_) of 999 mA/mm were measured from the OEP GaN HEMT with the 1 μm line patterns at V_DS_ = 10 V, which both demonstrated the highest value among the four OEP HEMTs, as shown in Table 2. The I_DSS_ is defined as the drain-to-source current (I_DS_) when the gate-to-source voltage (V_GS_) equals zero and the G_m, peak_ is defined as the peak extrinsic transconductance value of the device with a V_GS_ swept from −4 V to 0 V.

**Figure 4:**
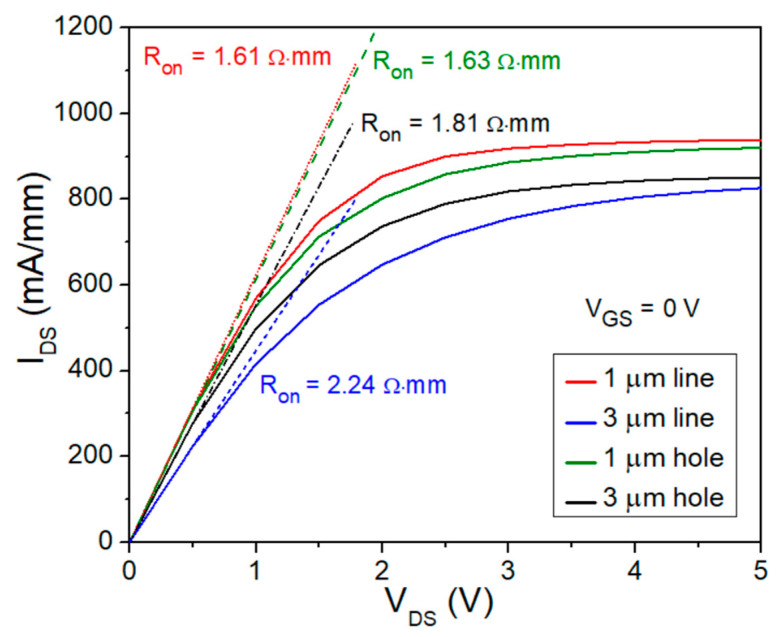
R_on_ and I_DS_–V_DS_ curves for the four 2 × 25 μm AlGaN/GaN OEP HEMTs.

The I_DS_−V_DS_ curves of the four OEP HEMTs with V_GS_ equals to 0 V and V_DS_ sweeping from 0 V to 5 V and their on-resistance (R_on_) values were also measured and calculated, respectively, as shown in Figure 4 and Table 2. The HEMT devices with the 1 μm line OEP structure has the R_on_ of 1.61 Ω·mm, which shows the lowest R_on_ among the four designed OEP HEMTs.

**Figure 5:**
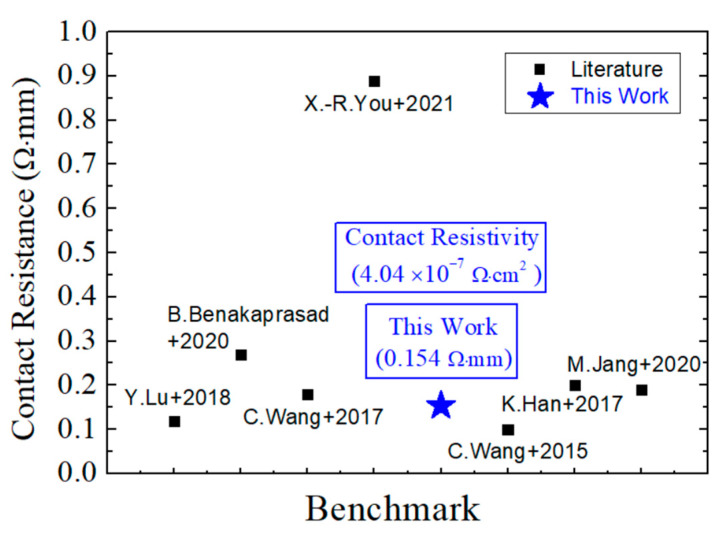
Benchmark of the lowest contact resistance in this study with published literature [20,21,22,23,25,26,27].

The measured DC characteristics of the four OEP HEMTs shown in Figure 3, Figure 4, Table 1, and Table 2 exhibit improvements in the device performance. The improvement of ρ_c_ from 2.73 × 10^−6^ Ω·cm^2^ to 4.04 × 10^−7^ Ω·cm^2^ and the improvement of R_c_ from 0.429 Ω·mm to 0.154 Ω·mm using the 1 μm line OEP structure is attributed to the increase of contact area at the interface between the ohmic metal stack and the semiconductor layer, forming more TiN_X_ layers and nitride vacancies, the inclusion of fringing effects, and the removal of irregular surface oxide layers [20,23,31]. Moreover, the increase in electron tunneling effect at the interface under the ohmic metal stack also stands a crucial role in the improvement of the ρ_c_ and R_c_ values and could be explained by the increase in N vacancies, increasing donor doping concentration and electric field, and thus increasing tunneling current [23]. A benchmark has been made to compare the lowest R_c_ in this work with well-known publications that also fabricated OEP GaN-based HEMTs, demonstrating the low R_c_ of the designed OEP HEMT in this study, as shown in Figure 5.

The trend of the G_m, peak_ and I_DSS_ are also analyzed for the four OEP HEMTs, showing that 1 μm line OEP HEMTs exhibit the highest values among the four designed structures, followed by the OEP HEMTs with the 1 μm holes, the 3 μm holes, and the 3 μm lines, in descending sequence. This is attributed to the larger contact area at the interface of the 1 μm line OEP HEMTs and that the 1 μm line OEP HEMTs still obtain enough AlGaN barrier layer to form enough 2DEG. R_on_ values of the four designed OEP HEMTs were also analyzed and demonstrated a similar trend to that of the I_DSS_ value. The trend of the R_on_ improvement for OEP HEMTs compared to non-OEP HEMTs was also found in previous research [32].

### 3.2. RF Characteristics

#### 3.2.1. Small Signal Performance

**Figure 6:**
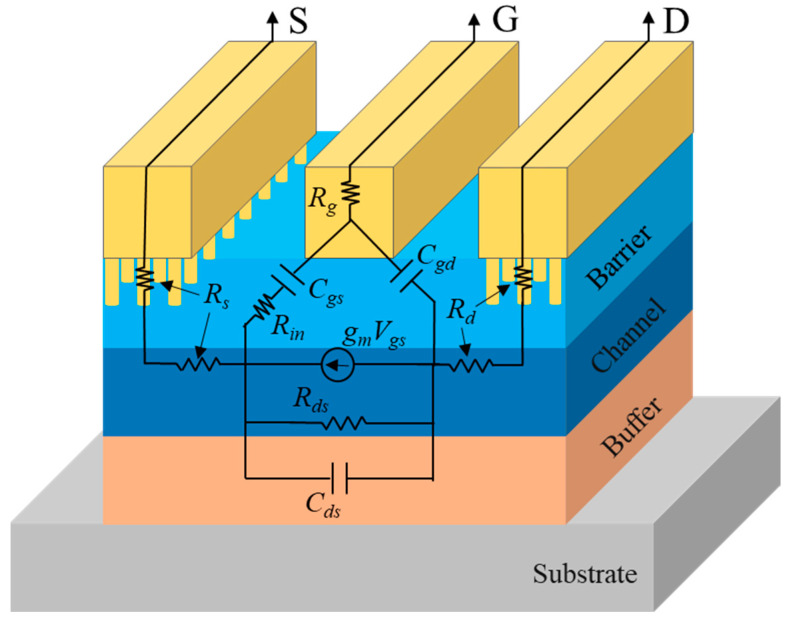
Small-signal equivalent circuit model for the AlGaN/GaN HEMTs with the hole OEPs.

**Figure 7:**
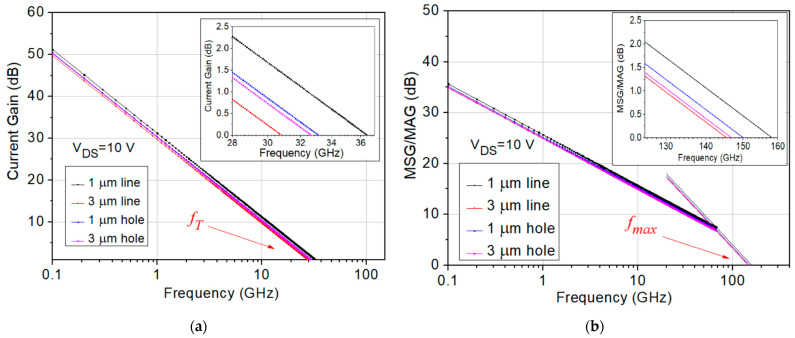
(**a**) Current gain to frequency plot and (**b**) MSG/MAG to frequency plot for the 2 × 25 μm OEP AlGaN/GaN HEMTs.

All the designed AlGaN/GaN OEP devices were measured with a E8361C PNA network analyzer and a 4142B DC supplier to obtain the S parameter results for small signal performance analysis. The small-signal equivalent circuit model for the OEP AlGaN/GaN HEMTs was used, as shown in Figure 6. The small signal impedance matching system was calibrated with a short-open-load-thru (SOLT) calibration with an accuracy of less than ±0.01 dB for both the S21 and S12 values and less than −45 dB for both the S11 and S22 values within the measured frequency range [33]. The measured S parameters were first de-embedded and the current gain (H21), maximum stable power gain (MSG), and maximum available gain (MAG) were calculated using the Microwave Office 2000 software. After extrapolating the H21 (dB) to frequency (log scale) curves and MSG/MAG to frequency (log scale) curves with the slope of −20 dB/decade, the cut-off frequency (*f_T_*) and maximum oscillation frequency (*f_max_*) values of the OEP devices were obtained, as shown in Figure 7a,b.

The de-embedded *f_T_* values of the designed 1 μm line, 3 μm line, 1 μm hole, and 3 μm hole OEP HEMTs are 36.40 GHz, 30.90 GHz, 33.10 GHz, and 32.60 GHz, respectively, as shown in Table 3. The de-embedded *f_max_* values of the designed 1 μm line, 3 μm line, 1 μm hole, and 3 μm hole OEP HEMTs are 158.29 GHz, 145.50 GHz, 150.05 GHz, and 146.80 GHz, respectively, as shown in Table 3. Among the four designed OEP HEMTs with the gate width of 2 × 25 μm, the *f_T_* and *f_max_* value of the 1 μm line OEP device exhibit the largest value of 36.4 GHz and 158.29 GHz, respectively.

The parasitic source resistance (*R_s_*), parasitic drain resistance (*R_d_*), parasitic gate-to-source capacitance (*C_gs_*), and parasitic gate-to-drain capacitance (*C_gd_*) of the four designed OEP HEMTs were also extracted from the S parameter results, as shown in Table 3. The 1 μm line OEP device exhibit the lowest *R_s_*, *R_d_*, *C_gs_*, and *C_gd_* of 4.35 Ω, 2.73 Ω, 91.03 fF, and 9.76 fF, respectively, among the four designed OEP HEMTs. The small signal results show that the 1 μm line OEP HEMTs exhibit the best small signal performance among the four designed structures, followed by the OEP HEMTs with the 1 μm holes, the 3 μm holes, and the 3 μm lines, in descending sequence.

The measured small signal characteristics of the four OEP HEMTs are shown in Figure 7 and Table 3. The results show that the 1 μm line OEP HEMT exhibited the highest *f_T_* and *f_max_* among other OEP HEMTs, which could be attrbuted to the reduction in parasitic resistances and parasitic capacitances. The equations showing the correlation between *f_T_*, *f_max_*, and the extracted parameters of *R_s_*, *R_d_*, *C_gs_*, and *C_gd_* are shown below in Equations (1) and (2) [34].

(1) $$f_{T}=\frac{g_{m}}{2\pi \left(C_{gs}+C_{gd}\right)\left[1+\left(R_{s}+R_{d}\right)g_{o}\right]+g_{m}C_{gd}\left(R_{s}+R_{d}\right)}$$

(2) $$f_{max}=\frac{f_{T}}{2\sqrt{g_{o}\left(R_{g}+R_{i}+R_{s}\right)+2\pi f_{T}R_{g}C_{gd}}}$$

Equations (1) and (2) show that the *f_T_* and *f_max_* value are inversely proportional to the parasitic components of *R_s_*, *R_d_*, *C_gs_*, and *C_gd_*.

The *R_s_*, *R_d_*, *C_gs_*, and *C_gd_* values of the four designed OEP HEMTs were analyzed and a similar ascending trend was found. The lowest parasitic values among the four designed structures were extracted from the 1 μm line OEP HEMTs, followed by the OEP HEMTs with the 1 μm holes, the 3 μm holes, and the 3 μm lines, in ascending sequence. This correlates to the trend of the *f_T_* and *f_max_* value measured from the four designed OEP HEMTs. The extracted *C_gs_* and *C_gd_* values of both the 3 μm hole and 3 μm line OEP HEMT are larger than the extracted *C_gs_* and *C_gd_* values of both the 1 μm hole and 1 μm line OEP HEMT, which shows that the increment in the size of the patterns from 1 μm to 3 μm increases the *C_gs_* and *C_gd_* values. The extracted *C_gs_* and *C_gd_* values of the hole OEP HEMTs also show larger values than the line OEP HEMTs, which is due to the increased separated ohmic metal arrays formed by the hole patterns. The increase in the contact area between the ohmic metal and the AlGaN barrier layer were used to reduce the contact resistance of the OEP HEMTs, and the results show that the 1 μm line OEP HEMT could be fabricated with the best improvement in small signal performance at the Ka band.

#### 3.2.2. Large Signal Performance

**Figure 8:**
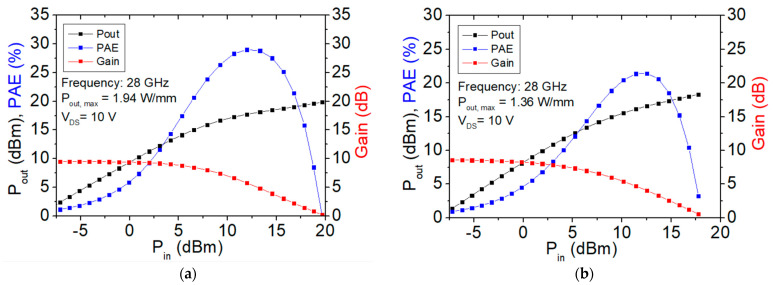

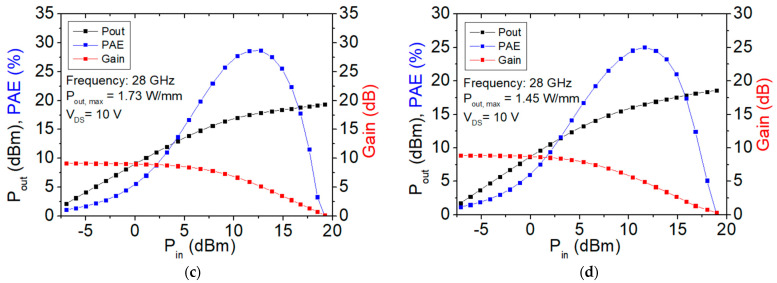
Load–pull curves at 28 GHz operation frequency for the 2 × 25 μm OEP AlGaN/GaN HEMTs with (**a**) 1 μm lines, (**b**) 3 μm lines, (**c**) 1 μm holes, and (**d**) 3 μm holes.

Load–pull measurements at 28 GHz operation frequency for RF power and PAE analysis were also conducted for the four designed OEP devices with a gate width of 2 × 25 μm. The power sweep curves of the load–pull measurement with input power set from −7.5 dBm to 20 dBm for the four designed OEP device structures with 1 μm lines, 3 μm lines, 1 μm holes, and 3 μm holes are shown in Figure 8a,b,c,d, respectively.

The peak PAE, the power gain, and the maximum output power density (P_out, max_) in units of dBm and W/mm of the four OEP HEMTs are shown in Table 4. The peak PAE of the 1 μm line, 3 μm line, 1 μm hole, and 3 μm hole HEMTs are 29.01%, 21.44%, 28.70%, and 25.03%, respectively. The power gain of the 1 μm line, 3 μm line, 1 μm hole, and 3 μm hole HEMTs are 9.52 dB, 8.60 dB, 9.12 dB, and 8.87 dB, respectively. The P_out, max_ (dBm) of the 1 μm line, 3 μm line, 1 μm hole, and 3 μm hole HEMTs are 19.86 dBm, 18.31 dBm, 19.36 dBm, and 18.60 dBm, respectively. The P_out, max_ (W/mm) of the 1 μm line, 3 μm line, 1 μm hole, and 3 μm hole HEMTs are 1.94 W/mm, 1.36 W/mm, 1.73 W/mm, and 1.45 W/mm, respectively. The load–pull results show that the 1 μm line OEP HEMTs exhibit the best large signal performance among the four designed structures, followed by the OEP HEMTs with the 1 μm holes, the 3 μm holes, and the 3 μm lines, in descending sequence.

The measured large signal characteristics of the four OEP HEMTs are shown in Figure 8 and Table 4. The 1 μm line OEP HEMTs exhibit the largest gain, PAE, and P_out, max_ among the four designed OEP HEMTs, followed by the OEP HEMTs with the 1 μm holes, the 3 μm holes, and the 3 μm lines, in descending sequence. The descending trend obtained from the large signal performance shown in Table 4 matches that of the DC characteristics and the small signal performances shown in Table 2 and Table 3, respectively. This could be due to the good thermal dissipation from the SiC substrate and low surface trapping of the OEP HEMTs with well deposited passivation layer [35]. The increase in the contact area between the ohmic metal and the AlGaN barrier layer was used to reduce the contact resistance and increase the saturation current of the OEP HEMTs, and the results show that the 1 μm line OEP HEMT could be fabricated with the best improvement in large signal performance at the Ka band.

#### 3.2.3. Noise Figure

**Figure 9:**
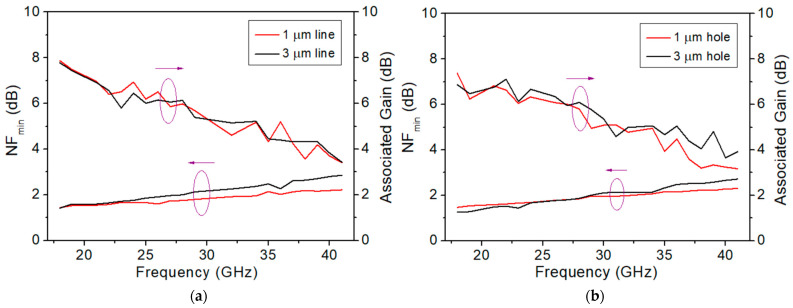
*NF_min_*- Frequency curves for the 2 × 25 μm OEP AlGaN/GaN HEMTs with (**a**) etched-line patterns and (**b**) etched-hole patterns. (Left arrow: *NF_min_*; right arrow: gain).

The noise figure measurement at the Ka band was carried out for all four OEP devices with a gate width of 2 × 25 μm. The frequency sweep for the noise figure measurement was set from 18 GHz to 41 GHz. The gain to *NF_min_* graphs of the line-etched and hole-etched devices are shown in Figure 9a,b, respectively. At 28 GHz, *NF_min_* of 1.75 dB with an associated gain of 5.98 dB and *NF_min_* of 2.00 dB with an associated gain of 6.14 dB were measured for the 1 μm line and 3 μm line OEP devices, respectively, as shown in Figure 9a and Table 2. At 28 GHz, *NF_min_* of 1.85 dB with an associated gain of 5.80 dB and *NF_min_* of 1.87 dB with an associated gain of 6.09 dB were measured for the 1 μm hole and 3 μm hole OEP devices, respectively, as shown in Figure 9b and Table 2. The results show that the OEP devices etched with 1 μm lines exhibit the lowest *NF_min_* among the fabricated devices with comparable associated gain.

The measured noise figure characteristics of the four OEP HEMTs are shown in Figure 9 and Table 2. The 1 μm line OEP HEMTs exhibit the smallest *NF_min_* among the four designed OEP HEMTs at 28 GHz, followed by the OEP HEMTs with the 1 μm holes, the 3 μm holes, and the 3 μm lines, in ascending sequence. The lowered *NF_min_* is due to the reduction of access resistance achieved from the thinned barrier layer at the ohmic patterns and the increased contact area at the ohmic metal and semiconductor interface, which reduce *R_s_* and *R_d_* [19]. However, larger areas of the etched-away barrier layers in the 3 μm line OEP devices cause larger depletion of the 2DEG and reduction in I_DS_, which further increase *R_s_* and *R_d_* [20]. The increased *R_s_* and *R_d_* in the 3 μm line OEP devices may also be the reason for the higher *NF_min_*, as shown in Equation (3). On the other hand, the parasitic *C_gs_* of the HEMT devices also plays an important role in determining the device *NF_min_* during high frequency noise figure measurement, as shown in Equation (3) [36]. The parasitic *C_gs_* values of the four designed OEP HEMTs are extracted, as shown in Table 3, and show a similar ascending trend to that of the ascending trend found in the measured *NF_min_* values of the four designed OEP HEMTs, as shown in Table 2.

(3) $${NF}_{min}=1+2\pi fK_{f}C_{gs}\sqrt{\frac{\left(R_{g}+R_{s}\right)}{g_{m}}}$$

The increase in the contact area between the ohmic metal and the AlGaN barrier layer was used to reduce the *R_s_* and *R_d_* of the OEP HEMTs, and the results show that the 1 μm line OEP HEMT could be fabricated with the best improvement in noise figure performance at the Ka band.

Further analysis comparing the device performance of GaN HEMTs with and without the 1 μm OEP structure could be pursued as future work. This analysis might involve exploring various ohmic etching depths to optimize contact resistivity.

## 4. Conclusions

The design and fabrication of AlGaN/GaN HEMTs with four different OEPs to optimize the Ka-band performances were discussed in this study. The 1 μm line, 3 μm line, 1 μm hole, and 3 μm hole OEP AlGaN/GaN HEMTs were analyzed with regard to DC and RF characteristics. Low ρ_c_ of 4.04 × 10^−7^ Ω·cm^2^ was also measured for the 1 μm line OEP HEMTs. Optimized G_m_ of 403 mS/mm and the I_DSS_ of 999 mA/mm were measured for the 1 μm line OEP HEMT. The small signal and large signal results of the OEP HEMTs were measured and the optimized performance achieved with the 1 μm line OEP HEMT. Moreover, the lowest *NF_min_* of 1.75 dB among four OEP HEMTs was achieved with the fabricated 1 μm line OEP HEMTs, showing improvement in the RF noise figure characteristics. Overall, the increase in the contact area between the ohmic metal and the AlGaN barrier layer were used to reduce the contact resistance of the OEP HEMTs, and the results show that the 1 μm line OEP HEMT could be fabricated with the best improvement in RF performance for future 5G and B5G system applications at the Ka-band.

## Figures and Tables

**Table 1:** Contact resistivity and contact resistance results of different OEP structures obtained with the TLM method.

Ohmic Etching Patterns	ρ_c_ (Ω·cm^2^)	R_c_ (Ω·mm)
1 μm line	4.04 × 10^−7^	0.154
3 μm line	7.80 × 10^−7^	0.212
1 μm hole	6.01 × 10^−7^	0.191
3 μm hole	7.68 × 10^−7^	0.199
w/o OEPs	2.73 × 10^−6^	0.429

**Table 2:** R_on_, G_m, peak_, I_DSS_, minimum noise figure at 28 GHz, and associated gain at 28 GHz of the 2 × 25 μm AlGaN/GaN HEMT devices with different OEP structures.

Ohmic Etching Patterns	R_on_ (Ω·mm)	G_m, peak_ (mS/mm)	I_DSS_ (mA/mm)	*NF_min_* at 28 GHz (dB)	Gain at 28 GHz (dB)
1 μm line	1.61	403	999	1.75	5.98
3 μm line	2.24	374	855	2.00	6.14
1 μm hole	1.63	393	932	1.85	5.80
3 μm hole	1.81	385	880	1.87	6.09

**Table 3:** *f_T_*, *f_max_*, and extracted small signal parameters of the 2 × 25 μm AlGaN/GaN HEMT devices with different OEP structures.

Ohmic Etching Patterns	*f_T_* (GHz)	*f_max_* (GHz)	*R_s_* (Ω)	*R_d_* (Ω)	*C_gs_* (fF)	*C_gd_* (fF)
1 μm line	36.40	158.29	4.35	2.73	91.03	9.76
3 μm line	30.90	145.50	5.04	3.43	94.84	10.82
1 μm hole	33.10	150.05	4.53	2.81	91.30	10.15
3 μm hole	32.60	146.80	4.75	2.93	93.42	10.56

**Table 4:** RF large signal load-pull measurement results of the 2 × 25 μm AlGaN/GaN HEMT devices with different OEP structures.

Ohmic Etching Patterns	PAE Peak (%)	Gain (dB)	P_out, max_ (dBm)	P_out, max_ (W/mm)
1 μm line	29.01	9.52	19.86	1.94
3 μm line	21.44	8.60	18.31	1.36
1 μm hole	28.70	9.12	19.36	1.73
3 μm hole	25.03	8.87	18.60	1.45

## Data Availability

Data are contained within the article.
